# Supplementary material for: Self-recovering passive cooling utilizing endothermic reaction of NH4NO3/H2O driven by water sorption for photovoltaic cell
Source: Nat Commun. 2023 Apr 25;14:2374. doi: 10.1038/s41467-023-38081-9 (PMC10130129; doi:10.1038/s41467-023-38081-9)
Supplement: Supplementary file 1 — Supplementary Information [file 41467_2023_38081_MOESM1_ESM.pdf]

Supplementary Information

Self-recovering passive cooling utilizing endothermic reaction of NH_4NO_3/H_2O driven by water sorption for photovoltaic cell

Seonggon Kim[1], Jong Ha Park[2], Jae Won Lee[3], Yongchan Kim[1,4], Yong Tae Kang[1,4*]

[1]Research Center for Plus Energy Building Innovative Technology, 145 Anam-ro, Seongbuk-gu, Seoul 02841, Republic of Korea

[2]Department of Mechanical Engineering, University of California, Berkely, Berkely, California 94720, United States

[3]Division of Mechanical Engineering, Korea Maritime & Ocean University, 727 Taejong-ro, Yeongdo-gu, Busan 49112, Republic of Korea

[4]School of Mechanical Engineering, Korea University, 145 Anam-ro, Seongbuk-gu, Seoul 02841, Republic of Korea

*Corresponding author

E-mail: ytkang@korea.ac.kr, Tel: +82-2-3290-5952

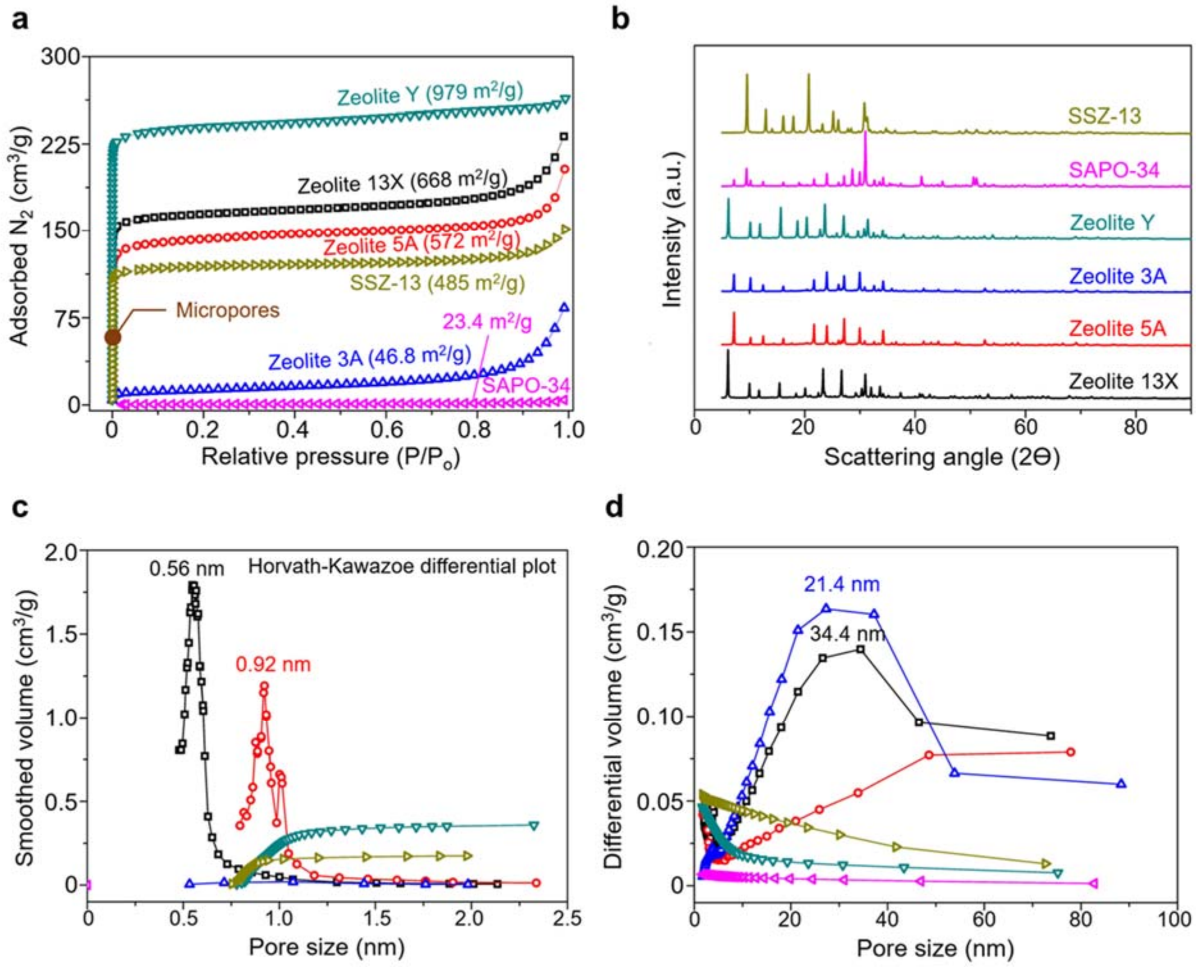

Fig. S1. Characterization of porous materials. **a** N_2 adsorption curve. **b** XRD pattern. **c** Micropore size distribution. **d** Mesopore size distribution. Source data are provided as a Source data file.

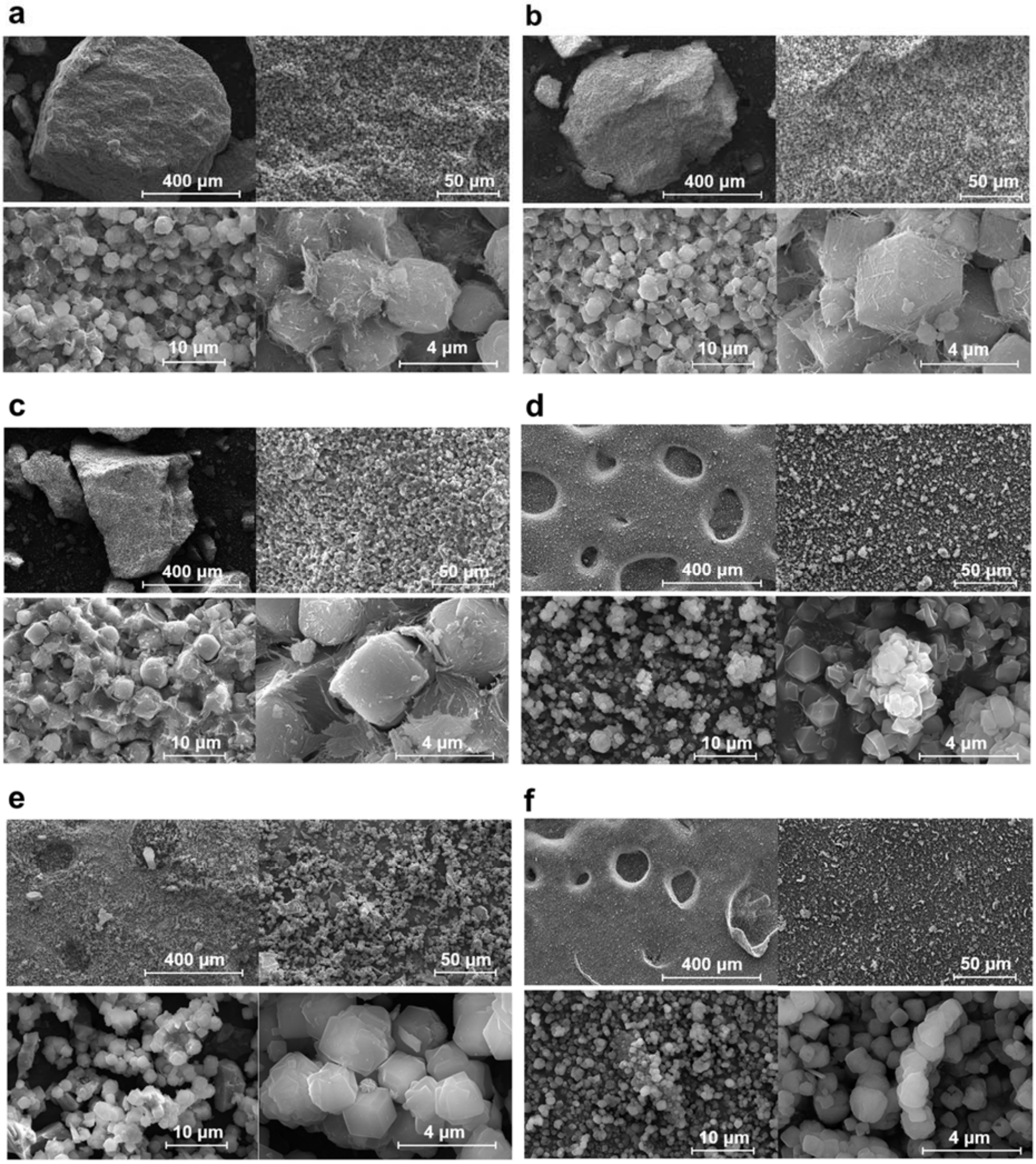

Fig. S2. FE-SEM images for porous materials. **a** Zeolite 13X. **b** Zeolite 5A. **c** Zeolite 3A. **d** Zeolite Y. **e** SAPO-34. **f** SSZ-13

Table S1. Components analysis using energy dispersive X-ray spectrometer

	Element (wt%)								
Materials	O	Na	Mg	Al	Si	Ca	K	Fe	P
Zeolite 13X	40.7	15.1	1.5	17.9	24.3	0.5	-	-	-
Zeolite 5A	41.9	2.6	0.9	18.9	22.1	13.6	-	-	-
Zeolite 3A	38.8	9.3	2.6	17.1	25.1	-	6.3	0.8	-
Zeolite Y	49.5	0.8	-	14.2	35.5	-	-	-	-
SAPO-34	39.0	19.0	0	20.8	20.0	-	-	-	1.2
SSZ-13	51.42	-	-	7.27	41.31	-	-	-	-

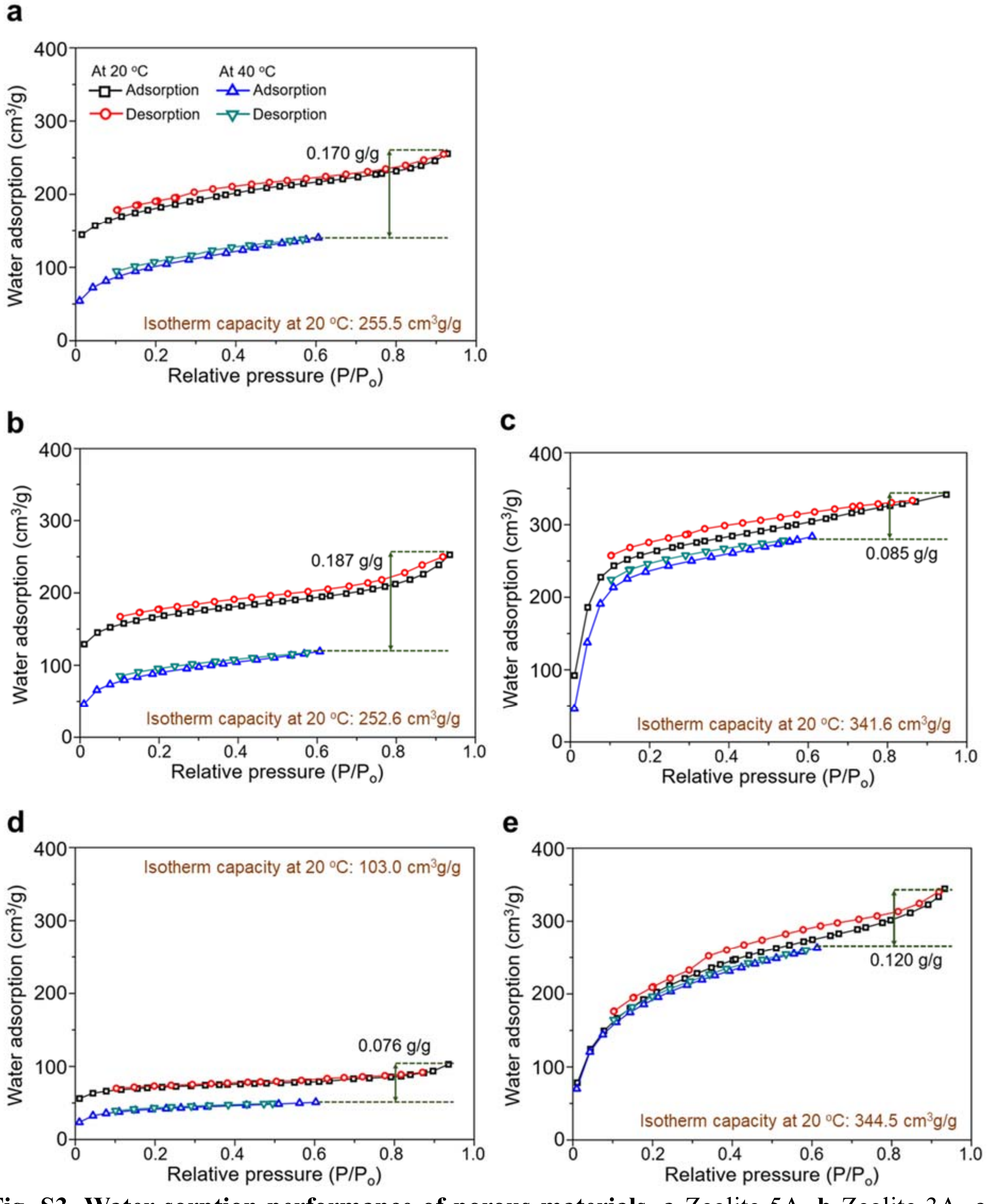

Fig. S3. **Water sorption performance of porous materials**. **a** Zeolite 5A. **b** Zeolite 3A. **c** Zeolite Y. **d** SAPO-34. **e** SSZ-13. Source data are provided as a Source data file.

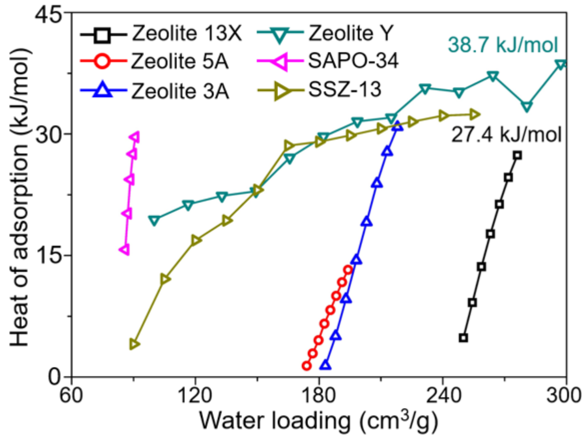

Fig. S4. Reaction heat versus water loading of porous materials. Source data are provided as a Source data file.

Table S2. Theoretical solubility difference in the range of 20-70 ^oC and endothermic reaction heat

Materials		Reaction heat (kJ/mol)	Solubility difference (g/g)
Solute	Solvent		
NH_4Cl	H_2O	14.76	0.92
NH_4NO_3	H_2O	28.05	3.96
KCl	H_2O	17.21	0.47
Na_2CO_3	CH_3COOH	26.48	0.022
$CoSo_4\cdot 7H_2O$	$SOCl_2$	90.82	0.87

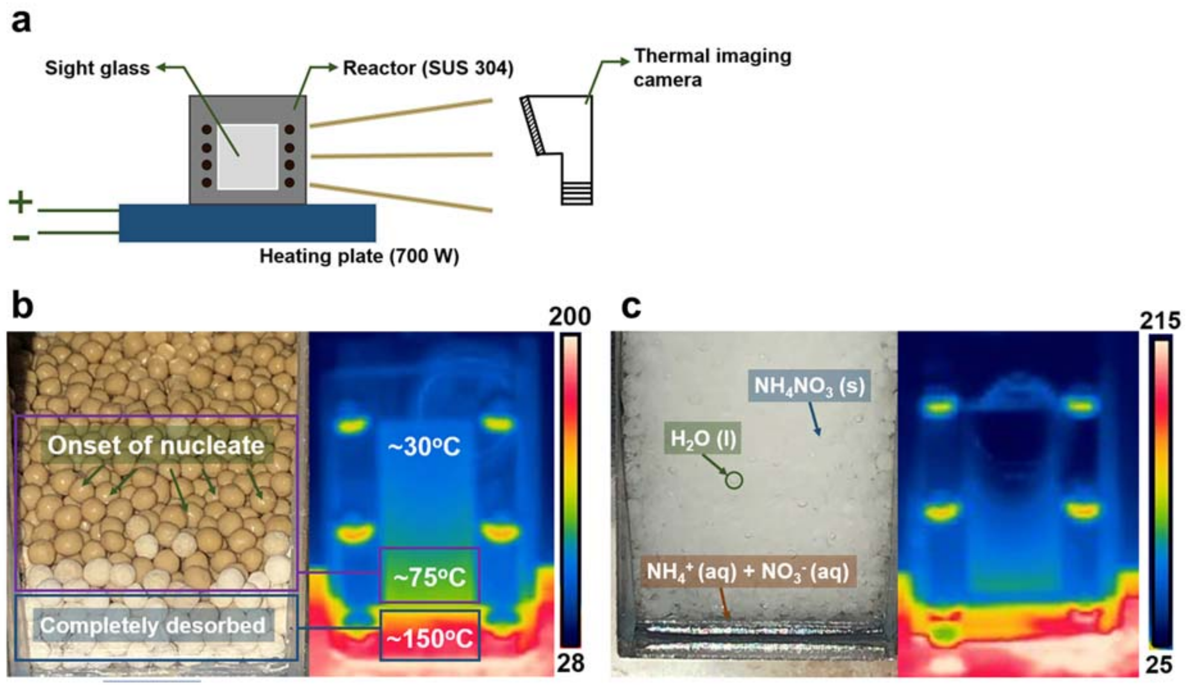

Fig. S5. **Visualization analysis during heat dissipation process**. **a** Schematic of thermal imaging experiment. **b** Latent cooling using zeolite 13X/H_2O. **c** Endothermic reaction cooling using NH_4NO_3/H_2O

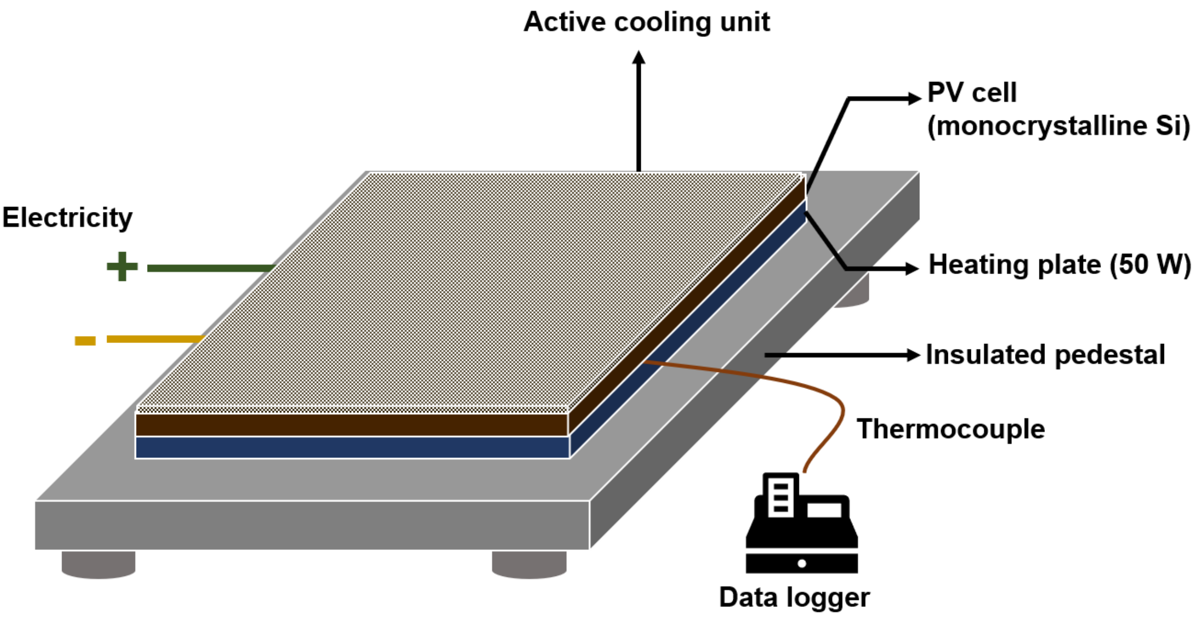

Fig. S6. Experimental device for evaluation of heat dissipation performances

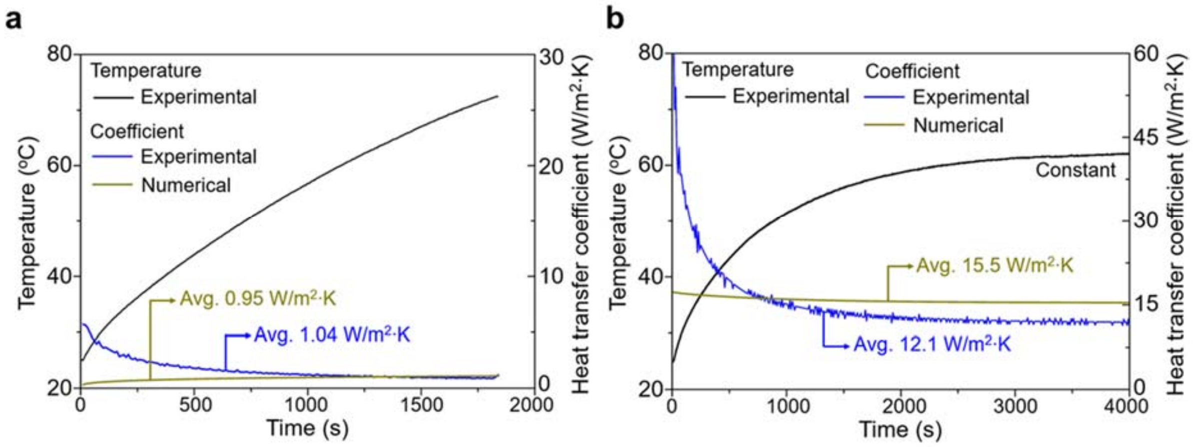

Fig. S7. Heat dissipation performance in terms of cooling methods. **a** Natural air cooling. **b** Forced air cooling. Source data are provided as a Source data file.

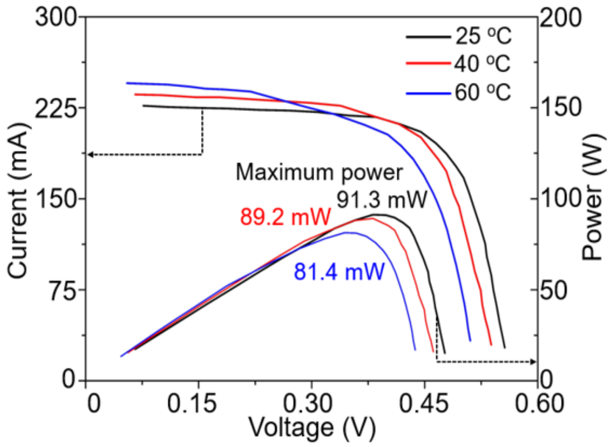

Fig. S8. Current-voltage characteristics in terms of temperature.

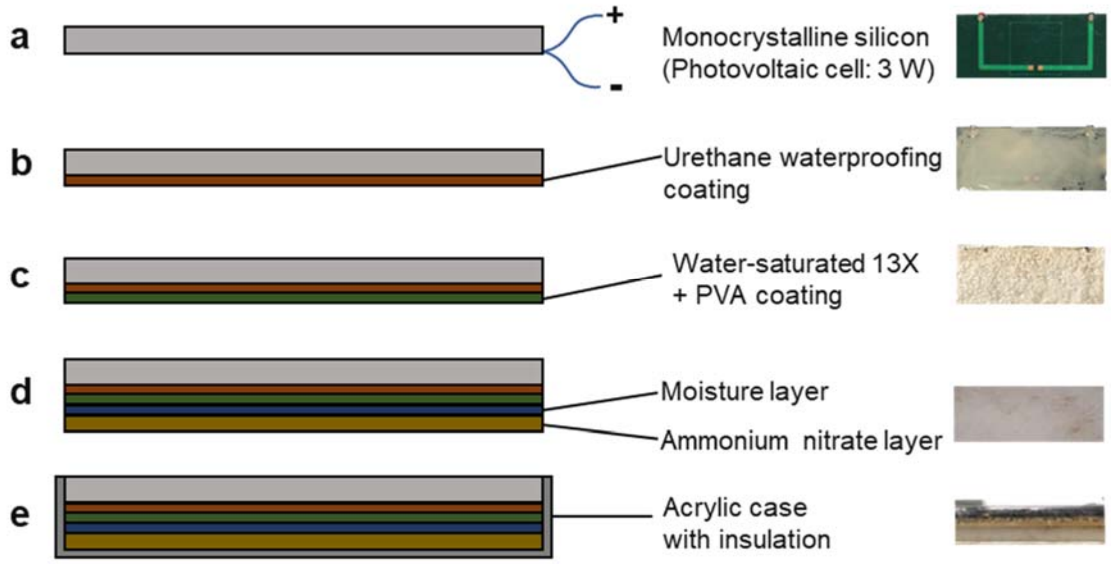

Fig. S9. Manufacturing method of PV cell with WD-ER cooling unit. **a** PV cell composed of monocrystalline silicon. **b** Waterproofing coating to protect power terminal unit. **c** Water-saturated zeolite 13X coating for latent cooling. **d** Ammonium nitrate coating for endothermic reaction cooling. **e** Sealing unit for safe operation.
