# Supplementary material for: Self-recovering passive cooling utilizing endothermic reaction of NH4NO3/H2O driven by water sorption for photovoltaic cell
Source: Nat Commun. 2023 Apr 25;14:2374. doi: 10.1038/s41467-023-38081-9 (PMC10130129; doi:10.1038/s41467-023-38081-9)
Supplement: Supplementary file 3 — Solar Cells Reporting Summary [file 41467_2023_38081_MOESM3_ESM.pdf]

nature research

Corresponding author(s): Yong Tae Kang

Solar Cells Reporting Summary

Nature Research wishes to improve the reproducibility of the work that we publish. This form is intended for publication with all accepted papers reporting the characterization of photovoltaic devices and provides structure for consistency and transparency in reporting. Some list items might not apply to an individual manuscript, but all fields must be completed for clarity.

For further information on Nature Research policies, including our data availability policy, see Authors & Referees.

Experimental design

Please check: are the following details reported in the manuscript?

1. Dimensions

Item	Yes	No	Details
Area of the tested solar cells	☒	☐	Methods, Practical applications
Method used to determine the device area	☐	☒	It is a commercially available product and has been verified with a vernier caliper.

2. Current-voltage characterization

Item	Yes	No	Details
Current density-voltage (J-V) plots in both forward and backward direction	☒	☐	Figure S8
Voltage scan conditions *For instance: scan direction, speed, dwell times*	☐	☒	This work has been focused on thermal management of PV cells
Test environment *For instance: characterization temperature, in air or in glove box*	☒	☐	Figure 6
Protocol for preconditioning of the device before its characterization	☐	☒	This work has been focused on thermal management of PV cells
Stability of the J-V characteristic *Verified with time evolution of the maximum power point or with the photocurrent at maximum power point; see ref. 7 for details.*	☐	☒	This work has been focused on thermal management of PV cells

3. Hysteresis or any other unusual behaviour

Item	Yes	No	Details
Description of the unusual behaviour observed during the characterization	☐	☒	There is no any unusual behavior
Related experimental data	☐	☒	There is no any unusual behavior

4. Efficiency

Item	Yes	No	Details
External quantum efficiency (EQE) or incident photons to current efficiency (IPCE)	☐	☒	This work has been focused on thermal management of PV cells
A comparison between the integrated response under the standard reference spectrum and the response measure under the simulator	☐	☒	This work has been focused on thermal management of PV cells
For tandem solar cells, the bias illumination and bias voltage used for each subcell	☐	☒	This work has been focused on thermal management of PV cells

5. Calibration

Item	Yes	No	Details
Light source and reference cell or sensor used for the characterization	☐	☒	This work has been focused on thermal management of PV cells
Confirmation that the reference cell was calibrated and certified	☐	☒	This work has been focused on thermal management of PV cells

Calculation of spectral mismatch between the reference cell and the devices under test

- [ ] Yes
- [x] No

This work has been focused on thermal management of PV cells

6. Mask/aperture

Size of the mask/aperture used during testing

- [ ] Yes
- [x] No

This work has been focused on thermal management of PV cells

Variation of the measured short-circuit current density with the mask/aperture area

- [ ] Yes
- [x] No

This work has been focused on thermal management of PV cells

7. Performance certification

Identity of the independent certification laboratory that confirmed the photovoltaic performance

- [ ] Yes
- [x] No

This work has been focused on thermal management of PV cells

A copy of any certificate(s)
Provide in Supplementary Information

- [ ] Yes
- [x] No

This work has been focused on thermal management of PV cells

8. Statistics

Number of solar cells tested

- [x] Yes
- [ ] No

One

Statistical analysis of the device performance

- [ ] Yes
- [x] No

There is no any statistical analysis of the device performance. This work has been focused on thermal management of PV cells

9. Long-term stability analysis

Type of analysis, bias conditions and environmental conditions
For instance: illumination type, temperature, atmosphere humidity, encapsulation method, preconditioning temperature

- [ ] Yes
- [x] No

This work has been focused on thermal management of PV cells.
